# Multiple Genome Sequences of Exopolysaccharide-Producing, Brewery-Associated *Lactobacillus brevis* Strains

**DOI:** 10.1128/genomeA.00585-17

**Published:** 2017-06-29

**Authors:** Marion E. Fraunhofer, Andreas J. Geissler, Frank Jakob, Rudi F. Vogel

**Affiliations:** Technische Universität München, Lehrstuhl für Technische Mikrobiologie, Freising, Germany

## Abstract

*Lactobacillus brevis* represents one of the most relevant beer-spoiling bacteria. Besides strains causing turbidity and off flavors upon growth and metabolite formation, this species also comprises strains that produce exopolysaccharides (EPSs), which increase the viscosity of beer. Here, we report the complete genome sequences of three EPS-producing, brewery-associated *L. brevis* strains.

## GENOME ANNOUNCEMENT

Beer represents a microbiologically stable beverage, as it combines a low pH and nutrient availability with the presence of hop compounds, ethanol, carbon dioxide, and anaerobicity (1). Nevertheless, certain lactobacilli are still able to grow in beer and spoil it. The resulting spoilage manifests as acidity, turbidity, off flavor, or increased viscosity. The latter is attributed to exopolysaccharides (EPSs) derived from members of the family *Lactobacillaceae* (2). To gain insights into this EPS synthesis, we sequenced the complete genomes of three brewery-associated* Lactobacillus brevis* strains.

*L. brevis* TMW 1.2108 and *L. brevis* TMW 1.2111 were isolated from wheat beer, and *L. brevis* TMW 1.2113 was isolated from a brewery surface. The Genomic-tip 100/G kit (Qiagen, Hilden, Germany) was used to isolate high-molecular-weight DNA from MRS liquid cultures. Single-molecule real-time (SMRT) sequencing (PacBio RS II) was performed at GATC (Konstanz, Germany) (3). For each of the three strains, a single library was prepared by selecting an insert size of 8 to 12 kb, resulting in at least 200 Mb of raw data from one SMRT cell (1 × 120-min movies), applying P4-C2 chemistry. The generated sequences were assembled with SMRT Analysis version 2.2.0.p2 using the Hierarchical Genome Assembly Process (HGAP) version 3 (4). The genome was completed by manual curation according to PacBio instructions and annotated using the NCBI Prokaryotic Genome Annotation Pipeline (PGAP) and the Rapid Annotations using Subsystems Technology (RAST) server (5–7).

Table 1 summarizes the characteristics, sequencing statistics, genome information, and accession numbers for each strain. The chromosome sizes range from 2.54 to 2.57 Mb, with G+C contents of 45.8% to 45.9%. The strains harbor 4 to 8 plasmids with G+C contents between 41.3% and 41.9% and sizes between 130.5 and 352.0 kb. The chromosomes encode five complete rRNA operons and 66 to 69 tRNAs.

**TABLE 1:** Strain characteristics, sequencing statistics, genome information, and accession numbers

Strain	Source	BioSample no.^a^	Accession no.^b^	Coverage (×)^c^	Size (Mb)	No. of contigs^d^	G+C content (%)	No. of CDSs^e^
*L. brevis* TMW 1.2108	Beer	SAMN04517635	CP019734 to CP019742	148	2.92	9	45.29	2,582
*L. brevis* TMW 1.2111	Beer	SAMN04517636	CP019743 to CP019749	341	2.88	7	45.32	2,440
*L. brevis* TMW 1.2113	Brewery surface	SAMN04517634	CP019750 to CP019754	364	2.67	8	45.74	2,357

a All BioSamples are part of BioProject no. PRJNA313253.

b Accession numbers are listed for all contigs of each whole genome (as a range).

c Average coverage of HGAP assembly.

d In the chromosomes plus plasmids and partial plasmids.

e CDSs, number of coding sequences (total) based on NCBI PGAP.

All strains possess a plasmid-encoded glycosyltransferase-2 (*gtf-2*), which has been described as a key enzyme for EPS synthesis of slimy, wine-spoiling members of the family *Lactobacillaceae* (8–10). Comparison of the *gtf-2* gene between the wine spoilers *Pediococcus parvulus* IOEB8801 (GenBank accession no. AF196967), *P. damnosus* 2.6 (GenBank accession no. AY999683), and* L. diolivorans* G77 (GenBank accession no. AY999684) and the beer spoilers reported here reveals sequence identities of 99%. This shows the *gtf-2* gene to be species-independent and highly conserved and might indicate a common origin.

The availability of these *L. brevis* genome sequences will allow a better understanding of EPS synthesis and its contribution to the spoilage of beer.

### Accession number(s).

The three complete *L. brevis* genomes have been deposited in DDBJ/EMBL/GenBank under the accession numbers given in Table 1.
